# The Role of Digital Workflow in Creating a New, Esthetic and Functional Smile in a Periodontally Compromised Patient: A Case Report

**DOI:** 10.3390/reports8030105

**Published:** 2025-07-08

**Authors:** Carlotta Cacciò, Marco Tallarico, Aurea Immacolata Lumbau, Francesco Mattia Ceruso, Milena Pisano

**Affiliations:** 1 Department of Medicine, Surgery and Pharmacy, University of Sassari, 07100 Sassari, Italy; carlotta.caccio@gmail.com (C.C.); alumbau@uniss.it (A.I.L.); milenapisano@yahoo.it (M.P.); 2 Department of Dentistry “Fra G.B. Orsenigo—Ospedale San Pietro F.B.F.”, 00189 Rome, Italy; f.m.ceruso@gmail.com

**Keywords:** aesthetics, BOPT, mandibular movements, CAD/CAM, smile creator, veneers

## Abstract

**Background and Clinical Significance:** Prosthetic rehabilitation in the aesthetic zone of periodontally compromised patients presents a complex clinical challenge, requiring a careful coordination of aesthetic, functional, and biological demands. This case highlights the benefits of digital dentistry, interdisciplinary collaboration, and regular maintenance in achieving long-term success in complex rehabilitations of periodontally compromised patients. **Case Presentation:** This case report describes the digital minimally invasive rehabilitation of a 39-year-old male patient with Stage III periodontitis, occlusal discrepancies, tooth mobility, and an interincisal diastema. A fully digital workflow—including intraoral scanning, aesthetic previewing, and mandibular motion analysis—was employed to guide diagnosis, treatment planning, and prosthetic execution. Conservative tooth preparations using a biologically oriented approach (BOPT) were combined with customised provisional restorations to support soft tissue conditioning and functional control throughout the provisional phases. Mandibular motion tracking facilitated the design of a personalised anterior guidance to improve occlusion and correct the deep bite. The interincisal diastema was initially maintained then closed during the advanced phase of treatment based on aesthetic simulations and patient preference. One unplanned endodontic treatment was required during the provisional phase, but no other complications occurred. **Conclusions:** At the four-year follow-up, the patient demonstrated stable periodontal and occlusal conditions, improved clinical indices, and high satisfaction with the aesthetic outcome.

## 1. Introduction and Clinical Significance

In recent decades, the advancement of digital technologies has markedly transformed clinical dental practice, fostering approaches that are increasingly predictable, minimally invasive, and tailored to individual patient profiles. The digitalization of clinical workflows has significantly influenced all domains of dentistry, including diagnostic protocols, treatment planning, and the fabrication of prosthetic, orthodontic, and surgical devices [1]. Within these evolving technologies, intraoral scanning [2], digital mandibular motion tracking [3], and aesthetic–functional previewing have emerged as integral components in enhancing both treatment quality and patient satisfaction. Intraoral scanners (IOS) have, in many instances, supplanted traditional impression techniques, offering a series of clinical and operational advantages. These include improved patient comfort, decreased distortion associated with conventional impression materials, real-time quality control of digital acquisitions, and seamless integration with CAD/CAM systems for prosthetic design [4,5,6,7]. Moreover, IOS technology constitutes the foundational element for the generation of a “digital twin” of the patient, facilitating advanced diagnostic simulations, analytical evaluations, and interdisciplinary planning. In addition to IOS, the integration of digital technologies for recording mandibular dynamics enables a precise assessment of functional occlusion [8,9], mandibular movement trajectories, and the craniomandibular relationship. These datasets are particularly relevant in cases involving temporomandibular disorders, full-mouth rehabilitations, or extensive restorative interventions, where vertical dimension, centric relation, and functional mandibular pathways critically influence prosthetic planning [10,11]. The dynamic registration provided by systems like Zebris supports individualised occlusal schemes that align with the patient’s natural biomechanics, thereby enhancing long-term functional outcomes and reducing the risk of complications.

A further key component of the digital workflow is aesthetic and functional previsualization. Software platforms allow clinicians to simulate final treatment outcomes by integrating facial and intraoral photographs with digital scans [12]. These tools enable a comprehensive evaluation of dental and facial proportions, incisal edge position, smile line, and gingival architecture, facilitating the validation of the proposed treatment plan before initiating any irreversible procedures. The use of digital smile design enhances interdisciplinary communication, improves patient understanding and engagement, and significantly contributes to case acceptance by allowing for a preview and modification of the aesthetic outcome based on objective criteria.

This case report presents an integrated fully digital approach to aesthetic and functional prosthetic rehabilitation, incorporating intraoral scanning, digital mandibular motion registration, and smile previewing technologies, in a young patient with stage III periodontitis. In the treatment of stage III periodontitis with predominant horizontal bone loss, residual pockets after non-surgical therapy represent a clinical challenge. The Minimally Invasive Non-Surgical Technique (MINST) offers an effective alternative to reduce sites with PD ≥ 5 mm and bleeding on probing, improving patient acceptance and reducing morbidity compared to conventional therapies. The objective is to demonstrate how the synergistic application of these tools contributes to more precise treatment planning, an enhanced predictability of prosthetic outcomes, and superior functional and aesthetic results. The case also emphasises the value of digital documentation in facilitating long-term follow-up and interdisciplinary collaboration.

## 2. Case Presentation

**Figure 1:**
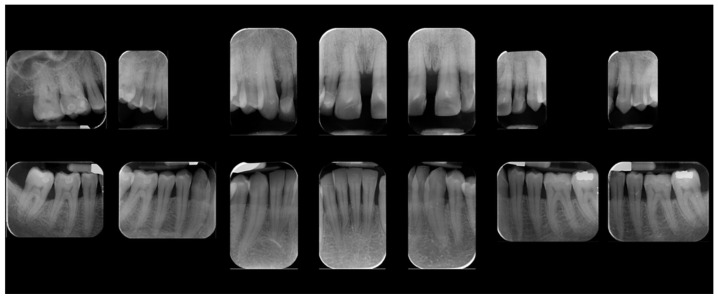
Initial radiographic assessment.

A 39-year-old male patient presented for prosthetic rehabilitation of the anterior maxillary region, primarily motivated by aesthetic concerns and periodontal health improvement. The patient reported a history of smoking (10–15 cigarettes per day since he was 18 years old) and had been diagnosed with localised stage III, grade B periodontitis. Even if the defect is mostly horizontal and localised to the II sextant, stage III was defined due to the bone loss extending to the middle third of the roots and the probing depth is ≥6 mm in this sextant. Moreover, smoking represents a risk factor able to classify the case as grade B. Initial clinical and radiographic evaluation (Figure 1) revealed the absence of all four third molars, the presence of conservative restorations on teeth 1.6, 3.7, and 4.6, and evidence of horizontal alveolar bone resorption in the regions corresponding to teeth 1.4 and 2.3. A conoid morphology was noted in the upper left lateral incisor.

**Figure 2:**
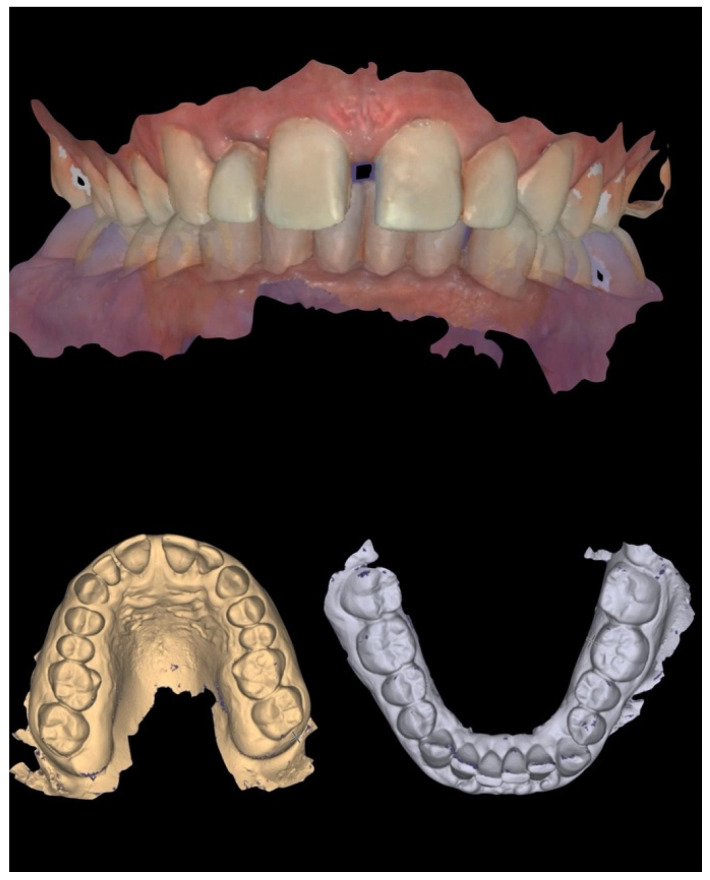
Initial intraoral scans.

**Figure 3:**
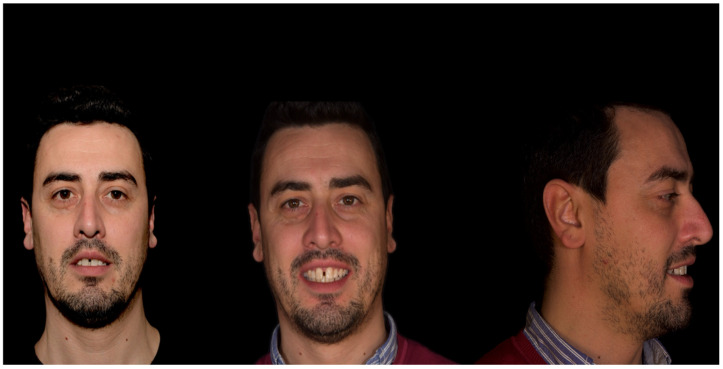
Initial extraoral photographs.

**Figure 4:**
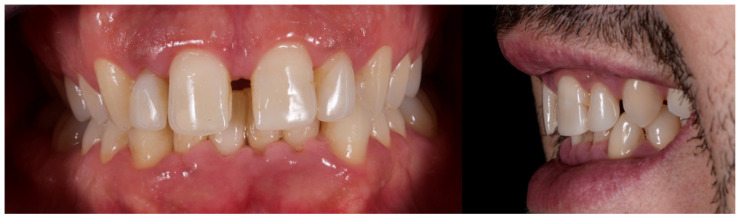
Initial intraoral photographs.

In addition to standard radiographs, a cone beam computed tomography (CBCT) scan was performed to obtain a three-dimensional assessment of the alveolar bone volume and morphology in the anterior maxillary region. Digital intraoral impressions were acquired using the Medit i500 scanner (MEDIT Corp., Seoul, Republic of Korea, Figure 2), complemented by intraoral and extraoral photographic records for comprehensive facial and dento-labial analysis (Figure 3 and Figure 4), as well as an aesthetic predictive simulation using digital smile design software (exocad’s DentalCAD 3.2 Elefsina, Smile Creator, exocad GmbH, Darmstadt, Germany).

**Figure 5:**
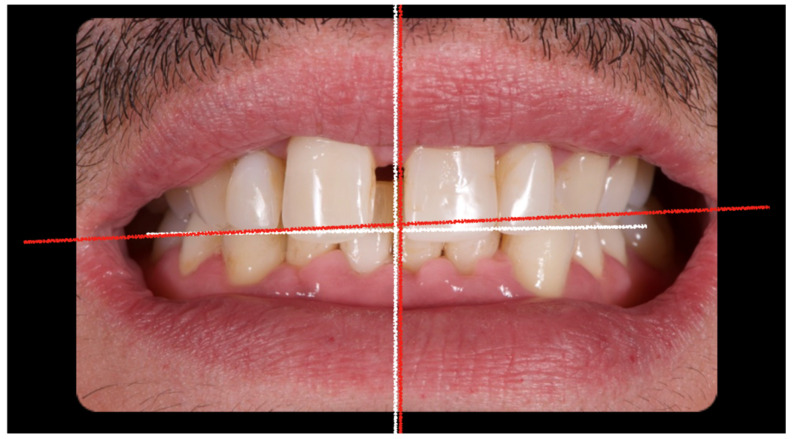
Clinical image demonstrating coincidence of the facial midline with the maxillary interincisal line. A midline diastema is evident.

The initial aesthetic evaluation revealed a 7 mm incisal display at rest, a flat incisal edge morphology, a high smile line, and a smile width encompassing ten teeth with normal buccal corridors. The facial midline was aligned with the interincisal midline, although a diastema was observed between teeth 1.1 and 2.1 (Figure 5).

**Figure 6:**
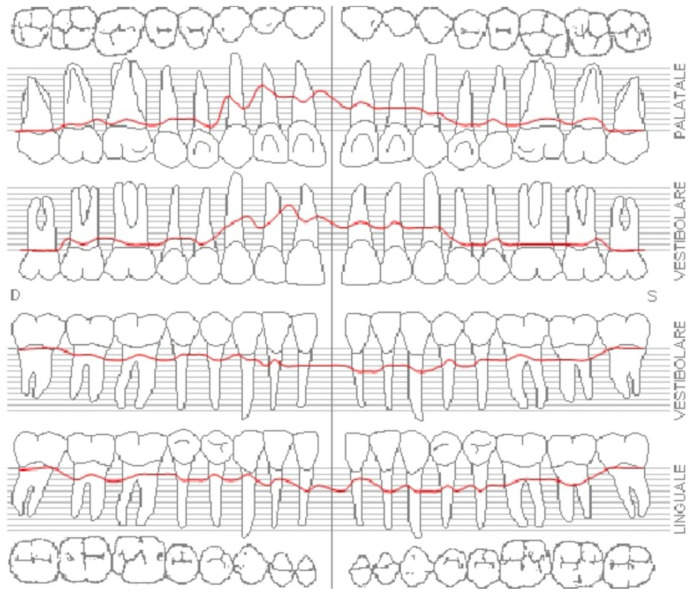
Initial periodontal examination. Red lines indicate the probing depth.

The occlusal plane appeared canted relative to both the labial commissure and the horizontal reference plane, whereas the interpupillary line was centred. Functional analysis demonstrated a minor discrepancy between the centric relation and maximum intercuspation (0.5 mm), a deep bite, and group function occlusion. An evaluation of maxillary positioning, mandibular dynamics, and temporomandibular joint (TMJ) function was conducted using the Zebris Jaw Movement Analysis System (JMA, zebris Medical GmbH, 88316 Isny, Germany). Periodontal assessment at baseline revealed stage III, grade B periodontitis with a mean probing depth of 2.6 mm, mean sextant probing depth of 4.8 mm, clinical attachment loss averaging 2.2 mm, a plaque index of 12%, bleeding on probing at 16%, and grade I mobility of teeth 1.1, 1.2, 2.1, and 2.2 (Figure 6).

Following the diagnostic evaluation and presentation of treatment alternatives, the proposed comprehensive rehabilitation plan was accepted.

1. Initial periodontal therapy.
2. First temporary restorations made up of a milled PMMA bridge from tooth 13 to 11 and a second one from tooth 21 to 22.
3. Endodontic therapy on tooth 22.
4. Second temporary restorations on definitive teeth preparations.
5. Definitive restorations made on porcelain fused to zirconia.
6. Maintenance periodontal therapy every four months.

**Figure 7:**
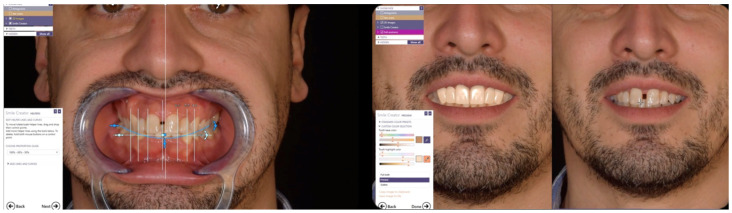
Laboratory workflow (CAD)—Smile Creator.

Initial periodontal therapy was performed to control inflammation and halt disease progression by removing supra- and subgingival plaque and calculus through professional cleaning (scaling and root planing), improving oral hygiene habits, and managing risk factors such as smoking and diabetes. A provisional restoration was then designed through a digital laboratory workflow (CAD—Smile Creator) (Figure 7) to simulate the final outcome and evaluate whether to maintain the interincisal diastema.

**Figure 8:**
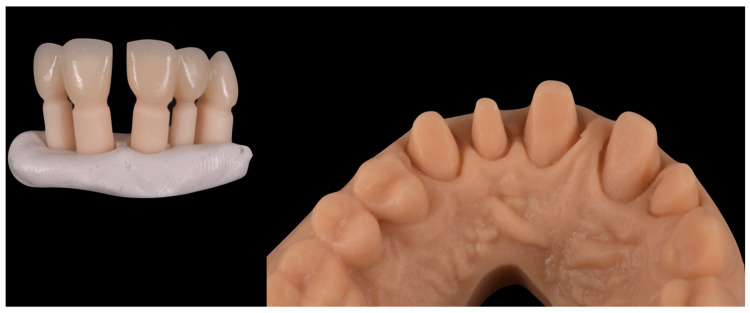
Laboratory workflow (CAD)—milled PMMA provisional restorations.

Since the patient declined orthodontic treatment, a pre-preparation provisional was fabricated to preserve the diastema, reduce overbite, and improve both anterior guidance and posterior group function. The initial extraoral photos with and without retractors were imported in exocad and converted to 3D objects, which can be matched to 3D scans of the teeth. Tooth shapes were selected from an extensive library and edited by using dedicated editing tools. Highly realistic AI-based (artificial intelligence) smile makeover visualisations (exocad’s DentalCAD 3.2 Elefsina, TruSmile Video, exocad GmbH) based on individual tooth setups and treatment plans were obtained and discussed with the patient. Provisional restorations were fabricated by CAD/CAM milling in polymethyl methacrylate (PMMA) (Figure 8).

**Figure 9:**
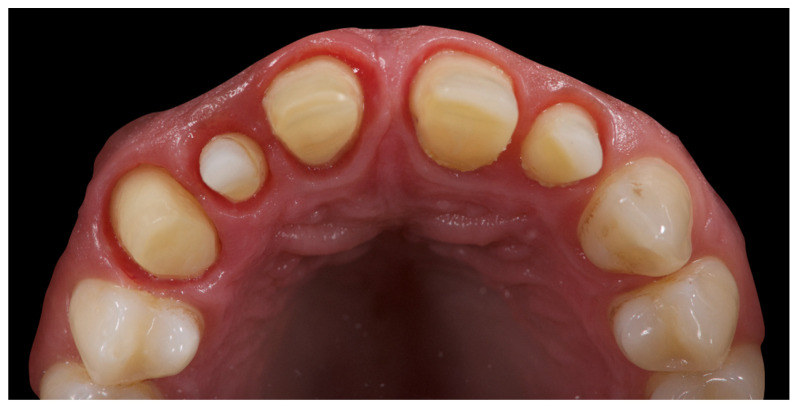
Initial tooth preparations.

Following the completion of initial periodontal therapy, edgeless tooth preparations were performed on teeth 1.3, 1.2, 1.1, 2.1, and 2.2 using a Komet 6863D rotary instrument (Komet Italia SRL, Verona, Italy): “Teeth have been prepared according to principles of the biologically oriented preparation technique (BOPT), preserving and supporting the periodontium.” (Figure 9). The BOPT involves removing the anatomical cemento-enamel junction (CEJ) without creating a defined finish line. Intentional gingival sulcus instrumentation (“gingitage”) causes a controlled bleed and clot formation. This promotes the reattachment of the junctional epithelium and connective tissue at a new prosthetic margin level. A provisional crown is relined and contoured with a concave emergence profile. It stabilises the initial clot and directs tissue shape, thickness, and migration during healing—effectively re-sculpting soft tissue architecture at the margin. Margin placement within the sulcus is carefully limited (≤1 mm) to avoid invading the biologic width and causing inflammation. This controlled vertical placement preserves healthy connective tissue and junctional epithelium. Vertical preparation also allows the technician to adapt the emergence profile post-preparation. The final crown margin becomes a prosthetic CEJ (PCEJ), supporting optimal periodontal adaptation and preserving tissue integrity.

**Figure 10:**
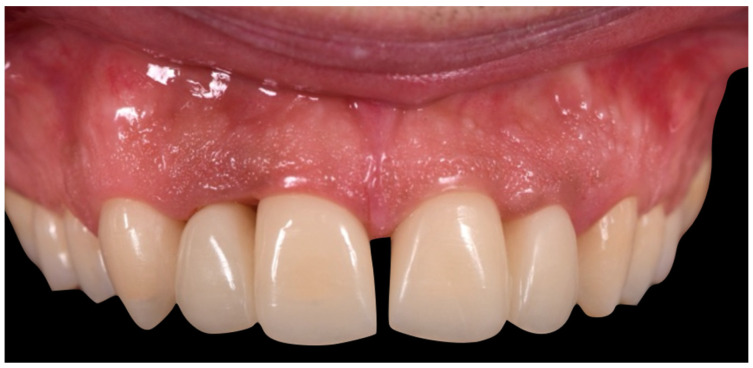
First provisional restoration.

The first set of provisional restorations (Figure 10) was cemented using a non-eugenol temporary luting agent (Temp-Bond™ Clear, Kerr Dental, 8302 Kloten, Switzerland).

**Figure 11:**
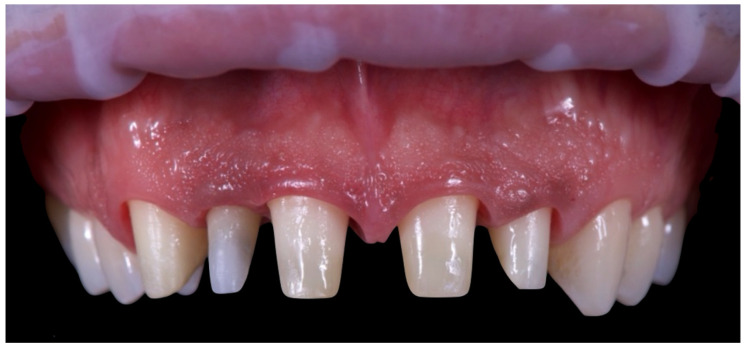
Final tooth preparations and microscopic refinement (16).

These provisionals were segmented into two units (right and left) and maintained the existing interincisal diastema. The deep overbite was corrected, and an appropriate incisal guide was established. Both provisional restorations were progressively modified every 2–3 weeks to promote soft tissue adaptation, manage the emergence profile, and support the re-establishment of a new prosthetic cementoenamel junction (CEJ) over time. This approach also allowed for the continuous evaluation of the patient’s phonetics and functional dynamics. Approximately four months later, the preparations were refined under 16× magnification to optimise the emergence profiles and support soft tissue conditioning while preserving the edgeless finish line to enhance papillary stability and control (Figure 11).

**Figure 12:**
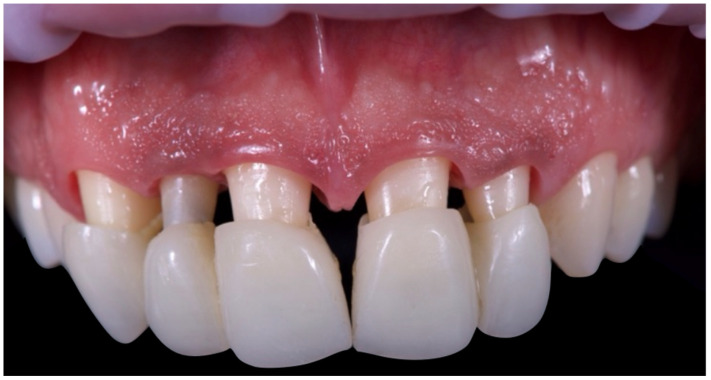
Second provisional restoration.

The second provisional restorations (Figure 12) were luted using zinc phosphate cement (Harvard) and mixed with petroleum jelly to facilitate later removal. At this stage, the interincisal diastema was closed. During the second provisional phase, tooth 1.2 developed symptoms of thermal sensitivity and mild pain, necessitating endodontic treatment.

**Figure 13:**
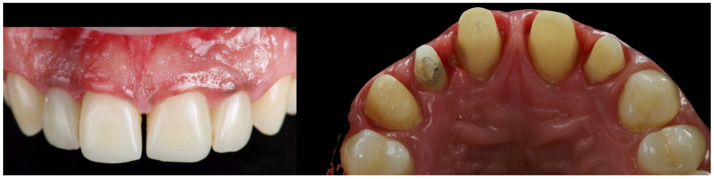
Nine-month follow-up.

**Figure 14:**
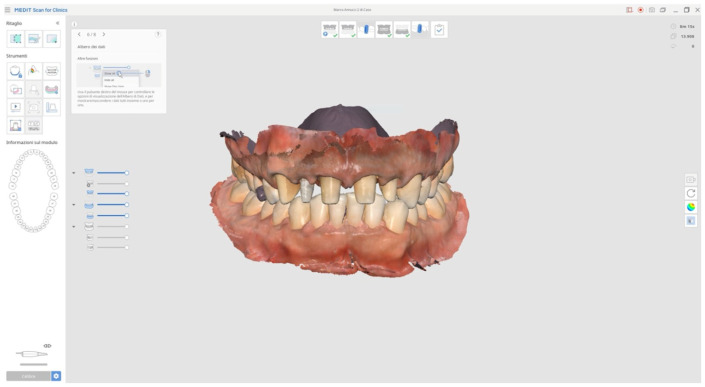
Final digital impressions taken with Medit Scanner (i500) using double retraction cord technique.

Due to restrictions related to the COVID-19 health emergency, the final impression phase was delayed and performed nine months later once soft tissue maturation had stabilised. Conventional impressions were obtained using the double-cord retraction technique (Figure 13 and Figure 14), followed by digital acquisition with the Medit i500 intraoral scanner (MEDIT Corp., MEDIT Corp, Seoul, Republic of Korea, Figure 14).

**Figure 15:**
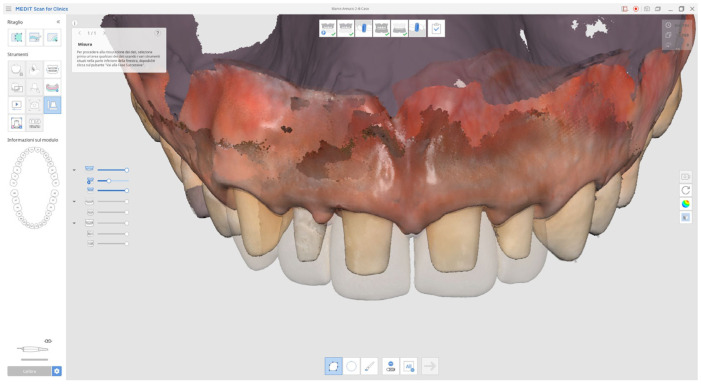
Superimposed digital scans of the preparations and provisional restorations (transparent area) for prosthetic assessment.

In addition, the provisional restorations were digitised, and mandibular movements, along with maxillary spatial orientation, were recorded using the Zebris Jaw Movement Analysis System (zebris Medical GmbH, 88316 Isny, Germany, Figure 15).

**Figure 16:**
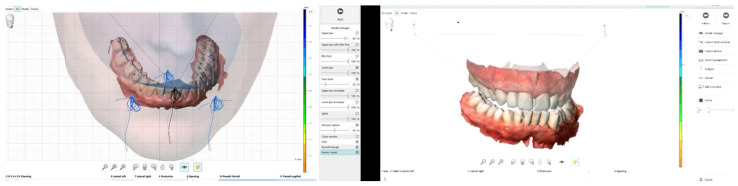
Mandibular movement analysis.

These records facilitated the individualised design of anterior guidance (Figure 16), calibrated to the condylar pathway in order to achieve functional posterior disclusion. A centric relation position with a 1 mm long centric stop was established, and the vertical overlap (overbite) was further reduced. A 1 mm long centric provided functional adaptability, reducing muscle and joint strain, and it also allowed for a smooth transition into anterior guidance without posterior interferences.

**Figure 17:**
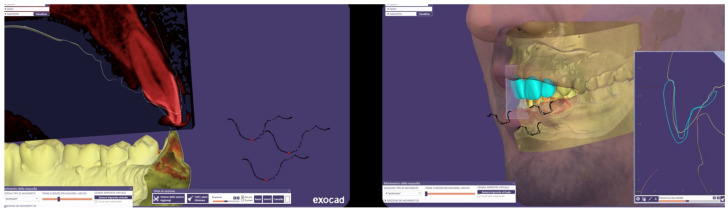
Anterior guidance development (laboratory phases).

A master model was generated upon which a monolithic zirconia framework was fabricated and subsequently layered with feldspathic ceramic. A clinical try-in was conducted to assess the passive fit, aesthetic parameters, and functional integration of the definitive restorations. Final cementation was performed using a conventional glass ionomer luting agent (Ketac Cem Easy Clean, 3M Italia SRL, Milan, Italy, Figure 17).

**Figure 18:**
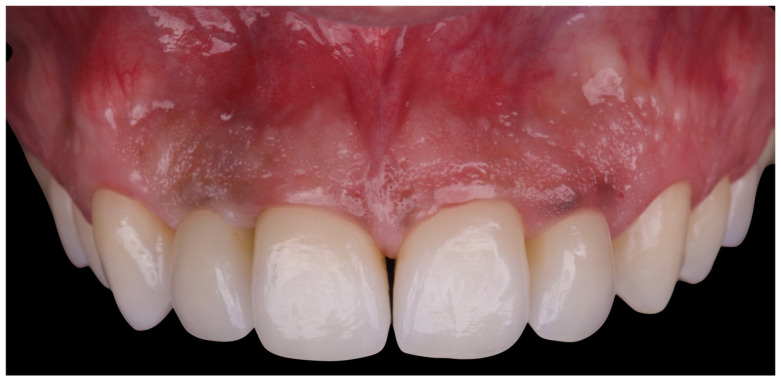
Final intraoral image.

**Figure 19:**
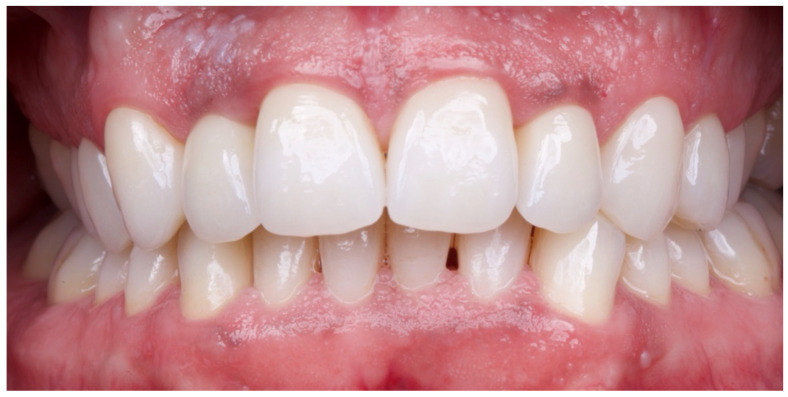
Intraoral image at four-year follow-up.

**Figure 20:**
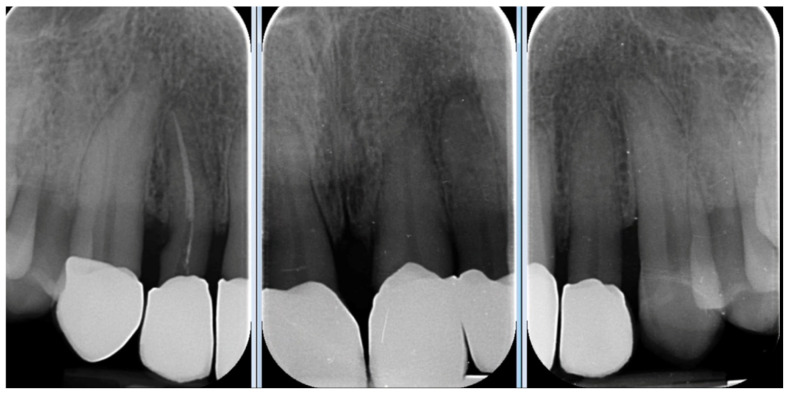
Four-year follow-up radiographic examination.

The patient was subsequently enrolled in a structured periodontal and prosthetic maintenance programme, with follow-up evaluations scheduled at four-month intervals to monitor periodontal health, occlusal stability, and the integrity of the prosthetic components (Figure 18, Figure 19 and Figure 20).

At the four-year follow-up, clinical evaluation demonstrated stable soft and hard tissues, with no biological or mechanical complications observed throughout the entire follow-up period.

## 3. Discussion

Prosthetic rehabilitation in the aesthetic zone of periodontally compromised patients presents a multifactorial clinical challenge, requiring the integration of aesthetic, functional, and biological considerations within a framework of risk management and long-term stability. In the present case, the presence of stage III periodontitis, occlusal discrepancies, tooth mobility, and an altered occlusal plane necessitated a comprehensive and minimally invasive treatment plan, supported by digital technologies and interdisciplinary collaboration. A key component of the approach was the strategic management of the interincisal diastema. Initially preserved in the first set of provisional restorations, the diastema allowed for gradual patient adaptation both functionally and aesthetically. Its subsequent closure during the advanced provisional phase was guided by aesthetic simulations using digital smile design software, which facilitated informed patient involvement and optimised treatment acceptance [12].

The overall digital workflow, incorporating intraoral scanning, mandibular motion tracking, and CAD/CAM design, proved essential in ensuring diagnostic accuracy and clinical predictability. These tools, some based on AI, allowed for high-precision execution, streamlined communication between clinical and laboratory teams, and enhanced control over prosthetic and occlusal parameters [2,8,9,10]. In particular, the use of digital mandibular motion registration enabled the creation of a customised anterior guidance scheme, improving posterior disclusion and contributing to the correction of the deep bite [10]. Tooth preparation followed the biologically oriented preparation technique (BOPT), employing edgeless margins to preserve the coronal structure and support gingival tissue stability. This approach is particularly advantageous in periodontally compromised patients, where interdental papilla preservation and emergence profile control are critical for long-term aesthetic success [13,14]. Customised provisional restorations were dynamically adapted over the course of treatment. Beyond their aesthetic function, they facilitated soft tissue conditioning, the refinement of occlusal relationships, and real-time monitoring of the periodontal response. Although one unplanned endodontic procedure was necessary due to post-operative sensitivity, no significant biological or mechanical complications occurred throughout the remainder of the treatment.

In the literature, similar cases have been reported, evaluating the role of fully digital workflows for the aesthetic rehabilitations of upper teeth. All the cases showed reduced clinical time, high accuracy, excellent aesthetic–functional outcomes, and mostly, the results were stable in the medium-term follow-up [15,16,17].

At the four-year follow-up, the clinical situation remained stable and asymptomatic. Periodontal evaluation revealed probing depths within physiological limits, a low plaque index (7%), and minimal bleeding on probing (8%), all consistent with a healthy periodontal environment. Radiographic assessment confirmed the maintenance of alveolar bone levels without signs of resorption. The patient’s reduction in tobacco use and adherence to four-month maintenance therapy intervals likely played a pivotal role in sustaining these outcomes. The four-year follow-up also confirmed the clinical stability of the treatment, both occlusally and periodontally, with a significant improvement in clinical indices and complete patient satisfaction. Masticatory function remained effective, the deep bite was corrected, and the interincisal diastema was successfully closed, resulting in a harmonious and stable aesthetic outcome. The final decision to fabricate individual crowns was justified by the favourable tissue response and the occlusal stability achieved.

The management of a periodontally compromised patient with high aesthetic demands required an interdisciplinary approach, grounded in careful planning and the integration of digital tools and conservative techniques. According to the S3-level clinical practice guideline, the treatment of stage III periodontitis should follow a structured stepwise approach—progressing from behavioural and risk factor management to non-surgical and surgical interventions—combined with long-term supportive care, all tailored to disease severity and supported by the best available evidence [18]. In the present case, non-surgical therapy and long-term maintenance have been combined. This approach is also supported by the 6-month results from a split-mouth randomised controlled trial, which showed that minimally invasive non-surgical therapy achieved clinical outcomes comparable to surgical treatment in patients with stage III periodontitis [19].

## 4. Conclusions

The clinical case presented demonstrated how the combination of targeted periodontal therapy, minimally invasive tooth preparations, digital occlusal registration, and customised CAD/CAM restorations can lead to predictable and durable aesthetic, functional, and biological outcomes. Take-home messages from this case underline the key factors contributing to the long-term success of complex prosthetic rehabilitations:

1. Regular follow-up to monitor tissue health and prosthetic integrity.
2. Strong patient compliance with oral hygiene and maintenance protocols.
3. Use of digital technologies to enhance precision, efficiency, and aesthetic outcomes.
4. Evidence-based preparation techniques to ensure gingival stability.
5. Multidisciplinary coordination for comprehensive patient-centred care.

## Data Availability

The original data presented in the study are included in the article, further inquiries can be directed to the corresponding author.
